# Exercise therapy in the treatment of tendinopathies of the lower limbs: a protocol of a systematic review

**DOI:** 10.1186/s13643-019-1058-9

**Published:** 2019-06-15

**Authors:** Yuri Rafael dos Santos Franco, Gisela Cristiane Miyamoto, Katherinne Ferro Moura Franco, Rodrigo Ribeiro de Oliveira, Cristina Maria Nunes Cabral

**Affiliations:** 1 Master’s and Doctoral Program in Physical Therapy of Universidade Cidade de São Paulo, Rua Cesário Galeno, 475, Tatuapé, SP Brazil; 2 Universidade Federal do Ceará, Rua Coronel Nunes de Melo, 1127, Fortaleza, CE Brazil

**Keywords:** Tendinopathy, Lower limb tendinopathies, Exercise therapy, Systematic review, Exercise prescription

## Abstract

**Background:**

Tendinopathies are specific degenerative conditions of the tendon characterized by pain and disability. The most common tendinopathies of the lower limbs are patellar, Achilles, gluteal, and proximal tendinopathy of the hamstring muscles. Exercise therapy has been studied for the treatment of these tendinopathies; however, different types of muscle contraction, exercise, dose, and intensity are found in the literature, which can make choosing the best treatment option difficult. The purpose of this systematic review is to analyze the available evidence about the effectiveness of exercise therapy in the treatment of patients with lower limb tendinopathies and the effects of different types of exercise therapy in the treatment of these patients.

**Methods:**

The search strategy will be performed in the following databases: CENTRAL, MEDLINE, EMBASE, PEDro, SPORTDiscus, and CINAHL. The inclusion criteria of the studies will be randomized controlled trials with patients with one of the following tendinopathies: patellar, Achilles, gluteal, and proximal tendinopathy of the hamstring muscles. The primary outcomes will be pain and disability. The intervention will be exercise therapy, and the comparators will be different types of exercise, control groups, or any other type of intervention.

**Discussion:**

Other systematic reviews have been published about the prescription of exercise therapy in the treatment of tendinopathies of the lower limbs. However, the results of these reviews are limited to only one type of tendinopathy or specific exercise. Because some of these reviews are also outdated, this systematic review will investigate whether exercise therapy is more effective than any other type of intervention and if there is a best form of exercise therapy, considering modality, dose, and intensity, for the treatment of lower limb tendinopathies. Furthermore, this study will present data related to the sample size, recruitment period, methodological quality, and visibility of the eligible studies.

**Systematic review registration:**

PROSPERO (CRD42018093011)

## Background

The tendon is the anatomical structure responsible for the transmission of force from the muscle to the bones [1]. Because of its role in supporting strength, the tendon is exposed to extreme conditions, which can lead to localized structural derangement [2]. Additionally, the tendon’s tissue receives little vascularization, has cells with low elasticity [1, 2], and shows decreased metabolism, being 7.5 times smaller than the other tissues of the locomotor system [2, 3]. Tendon dysfunction is called tendinopathy, which is characterized clinically by a reduction in the transmission of force from the muscle to the bone due to pain, thus generating disability in affected patients [4]. Tendinopathy does not have a specific etiology and may be connected to metabolic and mechanical conditions [2]. The metabolic conditions are related to diabetes, which influences the collagen quality of the tendon matrix [5–8], and obesity [9]. The mechanical conditions are associated with a much higher mechanical demand than the tendon can tolerate and are caused by movements such as jumping, landing, and abrupt change of direction, which are commonly adopted during lower limb sporting gestures [10, 11].

Among the common tendinopathies affecting the lower limbs, patellar tendinopathy is the most prevalent, especially in elite volleyball athletes [12], whose sporting gestures include jumping, landing, and constant direction change [12–14]. The second tendinopathy with a higher prevalence in the lower limbs, especially in runners (approximately 23% [12]), is Achilles tendinopathy, which is also related to the overload of local micro-trauma [15]. Proximal tendinopathy of the hamstring muscles still has an unknown prevalence, but the literature emphasizes that it is prevalent in sprinters [2, 16]. Similar to the others, proximal tendinopathy of the hamstring muscles is due to an imposed overload during training or recreational activity [16, 17]. Finally, gluteal tendinopathy affects about 45% of the population in developing countries and is unrelated to a specific work or sporting activity [18, 19]. Unlike the others, gluteal tendinopathy is most prevalent in patients with a low level of physical activity and may be a result of the overload of the gluteus medius, gluteus maximus, or both [18, 19].

The recommended intervention for lower limb tendinopathies should encompass the different etiological factors for a better resolution of the symptoms [20–22]. Current evidence shows that corticosteroid infiltrations present only short-term effects, and a prolonged use weakens the tissue favoring a tendon rupture [23, 24]. Electrotherapy is widely used in clinical settings, in which shock wave therapy is the most commonly used [25]. The results of the application of shock wave therapy are favorable, but the studies have a low methodological quality, which may influence the results [26, 27]. Laser therapy presents conflicting evidence that can be explained by the different parameters used considering time, energy, and potency [28]. When the use of laser therapy is combined with load exercise, the results are better for pain and disability than load exercise alone [29].

Exercise therapy is the resource with evidence of better methodological quality for the treatment of lower limb tendinopathies [30]. Among the different modalities of exercise, muscular strengthening exercises are the most researched [30–35]. However, the exercise therapy described in the literature is composed of different forms of muscular contraction [20, 21, 24, 33, 34, 36–43, 32, 35, 41, 44–46], doses [47, 48], and intensity [24, 32, 49], making it difficult for the clinician to choose the best treatment option.

Based on the literature, the prescription of exercise therapy to modulate the symptoms of patients with lower limb tendinopathies seems to be a good treatment option. However, it is still unclear if exercise therapy is the best option of treatment for patients with patellar, Achilles, gluteal, and proximal tendinopathy of the hamstring muscles, compared to other medical and physical therapy interventions. In addition, it is unknown if there is a better type of exercise or if the improvement of symptoms depends on the region of the tendinopathy. Thus, the purpose of this systematic review is to identify and summarize the available evidence about the effectiveness of exercise therapy in the treatment of patients with lower limb tendinopathies and the effects of different types of exercise therapy in the treatment of these patients.

## Methods

### Type of studies

Only randomized controlled trials already published will be included.

### Type of participants

#### Inclusion criteria

- People at least 18 years old with at least one of the lower limb tendinopathies (patellar, Achilles, proximal hamstring muscles, and gluteal)
- Patients who have undergone an exercise-based intervention

#### Exclusion criteria

- Patients with systemic metabolic diseases, even if controlled
- Patients with a history of tendon rupture
- Studies in which exercise is combined with other interventions and in which it is unclear whether the effect of the intervention is only from the exercise

### Types of interventions

The following exercise therapies will be included:

- Isometric, isotonic, and/or eccentric contractions
- Aerobic exercises
- Stretching exercises
- Functional and/or energy storage loading exercises

The comparators will be a waiting list, natural history of the disease, usual care, physiotherapeutic treatment based on other types of exercises, electrotherapy, thermotherapy or manual therapy, and medical treatment (use of oral medications and/or infiltration or surgical procedures, among others).

### Outcomes

All relevant clinical and biomechanical outcomes will be included. Physiological outcomes, such as pro-inflammatory serum levels and an increased number of collagen fibers or collagen type, will not be taken into account in this review.

#### Primary outcome

- Pain intensity measured by any reliable and valid instrument
- Disability measured by any reliable and valid instrument

#### Secondary outcome

- Disability assessed by any reliable and valid specific test
- Quality of life measured by any reliable and valid instrument
- Biomechanical outcomes such as range of motion, muscle activation, and muscle strength
- Histological and/or morphological alteration of the tendon
- Adverse effects

### Search strategies

Searches will be conducted without restrictions on language or date of publication in the following databases: CENTRAL (Cochrane Central Register of Controlled Trials, The Cochrane Library), MEDLINE (PUBMED), EMBASE (OvidSP, 1980 to 2018), Physiotherapy Evidence Database (PEDro), SPORTDiscus (EBSCO), and CINAHL (EBSCO). All databases will be evaluated from the date of creation. The search strategy will be based on the Cochrane Back and Neck Review Group for randomized controlled trials and systematic reviews about exercise therapy and tendinopathies. The search strategy will be divided into blocks: (1) interventions with different types of exercise therapy, (2) study design (randomized controlled trial), and (3) clinical condition (patellar tendinopathy, Achilles tendinopathy, proximal tendinopathy of the hamstring muscles, and gluteal tendinopathy). This protocol of systematic review was prospectively registered in PROSPERO (CRD42018093011).

### Data collection and analysis

#### Selection of studies

The selection of studies will be conducted by pairs of independent authors who will select titles and abstracts of possible eligible studies (YRSF/GCM) and will read the full text for final study inclusion (YRSF/GCM and KFMF/YRSF). If there is a disagreement, a third author will make the decision. The list of references of previous systematic reviews [20, 36] about lower limb tendinopathies, as well as the list of references of the eligible randomized controlled trials, will also be evaluated.

#### Data extraction and management

Data extraction from eligible studies will be performed by pairs of independent authors (YRSF/GCM and KFMF/YRSF) using a standard form that will be pretested with two randomized controlled trials on chronic patellar tendinopathy. If there is a disagreement between the data extracted, a meeting will be held to modify the decision of one of the authors. If the disagreement persists, a third author will arbitrate the decision. The data collected will be:

- Bibliometric (author, year of publication, and language)
- Characteristics of the studies (sample size, sample description, country, form of recruitment, recruitment time, and funding)
- Characteristics of participants (gender, age, height, weight, body mass index, affected size, and duration of symptoms)
- Description of the intervention (nature of the intervention, number of sessions, duration of each session, and presence of other interventions)
- Outcomes (when the total score of the outcomes is different, the values will be converted to a scale of 0 to 100 points)
- Results of the studies (mean and standard deviation or median and interquartile range and confidence interval)
- Duration of follow-up (short term up to three months, medium term up to six months, and long term up to 12 months or more, always after randomization [50])
- Altimetric values (will be collected directly from the website [18] of the article’s publication) [51]
- Score of the PEDro Scale (will be collected directly from the website https://www.pedro.org.au/) [52]

#### Methodological quality, risk of bias, and statistical report

This assessment will be based on the tool for assessment of methodological quality, risk of bias, and statistical report from PEDro, which is composed of 11 questions. However, only 10 questions related to the internal validity of the study are scored from 0 to 10, in which the higher the score, the better the methodological quality and statistical report of the study. The composition of the PEDro scale is as follows [52]: (1) eligibility criteria were specified; (2) subjects were randomly allocated to groups; (3) allocation was concealed; (4) the groups were similar at baseline regarding the most important prognostic indicators; (5) all subjects were blinded; (6) all therapists who administered the therapy were blinded; (7) all assessors who measured at least one key outcome were blinded; (8) measures of at least one key outcome were obtained from more than 85% of the subjects initially allocated to groups; (9) all subjects for whom outcome measures were available received the treatment or control condition as allocated, or, where this was not the case, data for at least one key outcome were analyzed by “intention to treat”; (10) the results of between-group statistical comparisons were reported for at least one key outcome; and (11) the study provided both point measures and measures of variability for at least one key outcome. Item 1 is not included in the final score.

For eligible studies without an available PEDro score, the assessment will be conducted by two independent authors (YRSF/GCM and KFMF/YRSF). If there is disagreement, a meeting will be held to solve the issue. If the disagreement continues, a third author will arbitrate the decision.

#### Measures of treatment effect

The measures used in the eligible studies are expected to be continuous so that we can determine the effect size for pain and disability. If possible, the results of these outcomes will be pooled and analyzed through the mean difference. The effect size will be defined in three distinct levels, in which a mean difference of less than 10% will be a small effect; between 10 and 20%, a moderate effect; and over 20%, a large effect [53].

If there are sufficient data, a meta-analysis will be performed subgrouping similar interventions for the primary outcomes. We will use the formal test for subgroup interaction of the Review Manager 5.3.

#### Quality of the evidence

The Grading of Recommendations Assessment, Development, and Evaluation (GRADE) approach will be used to assess the quality of the body of evidence with five aspects (study limitations, consistency of effect, imprecision, indirectness, and publication bias) for each outcome [54]. For these assessments, two assessors (YRSF/GCM) will independently rate the quality of the evidence using the GRADE approach with the following criteria to assign the grade of evidence [54]:

- High: We are very confident that the true effect lies close to that of the estimate of the effect
- Moderate: We are moderately confident in the effect estimate. The true effect is likely to be close to the estimate of the effect, but there is a possibility that it is substantially different
- Low: Our confidence in the effect estimate is limited. The true effect may be substantially different from the estimate of the effect
- Very low: We have very little confidence in the effect estimate. The true effect is likely to be substantially different from the estimate of the effect. In addition, this classification will be downgraded if one of the following items is identified [54]
  - Serious (− 1) or very serious (− 2) limitation of the study quality
  - Serious (− 1) or very serious (− 2) inconsistency
  - Some (− 1) or major (− 2) uncertainty about directness
  - Serious (− 1) or very serious (− 2) imprecise or sparse data
  - High probability of reporting bias (− 1)

## Discussion

The current literature shows that strengthening exercises are effective for the treatment of patients with lower limb tendinopathies [30, 31, 36]. However, consensus about the best modality of muscle contraction (isometric, isotonic, and/or eccentric), dose, and intensity of these exercises is absent, along with evidence of the effectiveness of stretching and balance exercises for the treatment of these patients. Thus, this review aims to reach clinicians for whom we will elucidate the best modality of exercise therapy for the different lower limb tendinopathies, with the possibility of determining the best dose and intensity. In addition, this review will elucidate methodological issues related to the sample size of the eligible studies and questions related to the recruitment period as well as assess the methodological quality of the existing studies, exploring important items that were not observed in the design of the eligible studies.

This systematic review will also address a new aspect related to the publication of studies, using Altmetrics. This assessment allows the measurement and monitoring of the extent and impact of studies and research through online interactions and will show the impact that each of the studies has had since publication [55].

## Data Availability

The datasets that will be used and/or analyzed during the current study will be available from the corresponding author on reasonable request.

## Abbreviations

CENTRAL: The Cochrane Central Register of Controlled Trials
CINAHL: Cumulative Index to Nursing and Allied Health Literature
EMBASE: Excerpta Medica dataBASE
GRADE: Grading of Recommendations Assessment, Development, and Evaluation
MEDLINE: Medical Literature Analysis and Retrieval System Online
PEDro: Physiotherapy Evidence Database
PROSPERO: International prospective register of systematic reviews
